# From “What the Hell Is Going on?” to the “Mushy Middle Ground” to “Getting Used to a New Normal”: Young People’s Biographical Narratives Around Navigating Parental Dementia

**DOI:** 10.1177/1054137316651384

**Published:** 2016-05-26

**Authors:** Mel Hall, Pat Sikes

**Affiliations:** 1 University of Sheffield, Sheffield, UK

**Keywords:** children, interviews, research methods, bereavement, illness

## Abstract

The number of young people who have a parent with dementia is rising as a result of improvements in diagnosis of young onset variants and demographic shifts. There has, however, been very little research focusing on this group. Accounts elicited as part of the Perceptions and Experiences of Young People With a Parent With Dementia described the period, usually some years, leading up to a diagnosis of a dementia and then the progress of the condition post diagnosis. These narratives were characterized by confusion, uncertainty, trauma, and distress as the young people struggled to make sense of the significant and often extreme, behavioral and attitudinal changes that were symptoms of the illness. This article describes and discusses how the young people experienced and navigated the temporal messiness and consequent biographical disruption arising from parental dementia.

## Introduction

While within the public perception, it would seem that dementia is equated with Alzheimer’s Disease causing memory loss in older people (Sikes and Hall, 2016); dementia is an umbrella term applied to a range of conditions which can both strike at different ages and affect the brain in various ways. These conditions include fronto-temporal dementia, vascular dementia, dementia with Lewy bodies, posterior cortical atrophy, Creutzfeld-Jakob disease, and dementias associated with Parkinson’s disease, HIV/AIDS and alcoholism. The symptoms of the different types of dementia can involve “prominent and disturbing behavioral changes” (Stewart, 2006) such as aggression, social withdrawal, loss of empathy, exhibitionism, short temper, obsessive compulsions, difficulties with planning and judgment making, visual disturbances, hallucinations, loss of physical functions, and aphasia and other communication disorders. A medical diagnosis is usually reached long after the initial symptoms present, not least because, and for a number of reasons, there tends to be a substantial delay between the first signs of noticing something is amiss and professional help being sought (Perry-Young, Owen, Kelly, & Owens, 2016; van Vliet et al., 2011). Once begun, the diagnostic process generally entails an initial consultation with a GP, followed by a series of tests and then a referral to specialists. This is a notoriously complicated and lengthy journey which typically stretches over a number of years. Studies report different average times—from 8 to 52 months—from first symptoms to first encounter with a health professional (Perry-Young et al., 2016) and then from there to diagnosis can also be variably prolonged (Chrisp, Thomas, Goddard, & Owens, 2011; Speechly, Bridges-Webb, & Passmore, 2008). The process can take even longer: (a) when the dementia is of a rarer type and is infrequently encountered and recognized (Crutch et al., 2013; Stewart, 2006) and (b) in cases of young onset dementia because relatives and clinicians may not initially consider the possibility of dementia (van Vliet et al., 2011).

It is estimated that dementia currently affects 850,000 people in the UK, of whom 42,325 are described as having a diagnosis of young onset dementia (YOD), that is, occurring before the age of 65. These numbers are set to increase due to longer life expectancy and advances in medical knowledge with incidence predicted to double to 2,092,945 by 2051 (Alzheimer’s Society, 2014).

The social and behavioral manifestations of dementia and the duration of the diagnosis pathway mean that dementia involves a great deal of uncertainty for all concerned, including family members (Teel & Carson, 2003). The rise in YOD diagnoses, coupled with social trends such as increased childbearing ages and the rise in second and subsequent families, means an increase in the numbers of children and young people living with a parent with dementia. Little is known, however, about how the condition affects these groups. Previous research has intimated that there are serious and profound social, psychological, and relationship consequences (Allen, Oybode, & Allen, 2009; Barca Thorsen, Engedal, Haugen, & Johannessen, 2014; Gelman & Greer, 2011; Harris & Keady, 2004; Hutchinson, Roberts, Kurrie, & Daly, 2014; Johannenssen et al., 2015; Luscombe, Brodaty, & Freeth, 1998; Millenaar et al., 2014; Roach et al., 2013; Robertson, 1996; Svanberg, Stott, & Spector, 2010; Svanberg, Stott, & Spector, 2011; van Vliet et al., 2011), but the evidence base is largely informed by adult children and grandchildren of people diagnosed after the age of 65 (Celdron, Triado, & Villar, 2011, 2012; Harper & La Fontaine, 2010). Green and Kleissen (2013) and Roach, Keady, Bee, and Hope (2008) have highlighted a need for direct research with children and young adults. Our study sought to address this need by taking a narrative auto/biographical approach to explore the perceptions and experiences of children and under 25 year olds who have or have had a parent with dementia.

## Methods

### Study Design

An approach that directly engaged with the young people’s perspectives was required. Auto/biographical and narrative, specifically life history, methodologies were consistent with this aim. Such approaches are appropriate for research committed to exploring lived experiences and are particularly useful in “giving voice to silenced lives” that is, to those whose accounts have not previously been sought or heard (McLaughlin & Tierney, 1993; Plummer, 2001; Goodson & Sikes, 2001; Goodson, Sikes, Andrews, & Atinkin, 2016). Rather than seeking to generalize, the aim is to gain a sense of how individuals with different biographies go through similar social and cultural experiences: in this case, being a young person with a parent who has dementia. Participants are “simply” invited to share their story of their parent’s dementia with the intention being to elicit detailed accounts about what the illness has meant for them.

The stories people tell are then considered in the particular social, historical, and other contexts in which they are lived, thereby helping to capture something of the complexity of lives and providing evidence to show how individuals negotiate their identities, construct futures, and make sense of social roles and rules. Analysis takes a thematic approach whereby themes are drawn out of narratives in a fashion that retains the story in its entirety (Riessman, 2008).

### Data Collection

Interviews were conducted with 22 children and young people aged 7 to 31, with the majority being between 16 and 24. This age span is in keeping with the United Nations and WHO definitions of youth (15–24). Despite our original intentions to speak to people between 7 and 25, we included 4 older participants because they had approached us, eager to share their experiences. We felt it would have been unethical to exclude them, and in any case, their retrospective accounts were clearly relevant.

The study was advertised by the Alzheimer’s Society (2014) and Young Dementia UK (2015) and broadcast across social media (Facebook and Twitter). A project website provided further information. This meant that the research came up on Internet searches, enabling some participants to find the study while searching for support. Ethical approval was granted by University of Sheffield.

Involvement entailed two to three interviews, ranging from 45 minutes to 2 hours, over a 16-month period thus capturing changes in circumstances over time.

The sample incorporates young people with parents with varying diagnoses and at different points along the dementia “journey”, for example, mid or recent diagnosis, years post diagnosis and death. Living circumstances also varied, with some living in the family home, others who lived elsewhere, and some whose parent was in residential care. We acknowledge the limitations of the study in that the sample was self-selecting and the majority of participants were White, British, and middle class. Table 1 provides specific detail.

**Table 1.** Overview of Participants.

Participant	Gender	Age	Parent	Diagnosis (if known)	Estimated years of dementia*	Family configuration	Living arrangements	Current activities
Sheena	Female	26	Dad	Dementia with Lewys Bodies	8	Youngest of 3 siblings	Away	Employed
Alex	Male	24	Dad	Frontal lobe dementia	4	Siblings	Away	Employed
Anna	Female	30					Away	Employed
Lily	Female	24	Mum	Early Onset Dementia (EOD)	2	Only child; divorced parents.	Away	Employed
Evie	Female	17	Dad	Fronto Temporal Dementia (FTD)	5	Only child	Father in a care home	Education
Elizabeth	Female	28	Mum	EOD	6	Youngest of 3 siblings	Away	Employed
Madison	Female	23	Mum	Posterior cortical atrophy	5	Only child; divorced parents	Away	Undergraduate university
Gabrielle	Female	23	Dad	Vascular dementia. Deceased 8 years ago (cancer related).	1	Parents divorced. Half sister from her father’s side.	N/A	Employed
Rachael	Female	24	Dad	EOD	2	Oldest of two siblings.	Away	Employed
Bethany	Female	21	Mum	FTD	6 Deceased 1 year ago.	Youngest of two siblings.	N/A	Postgraduate education
Grace	Female	31	Mum	EOD	10 (when aged 11–21). Mum passed away when Grace was 21.	Only child.	N/A	Employmed
Colin	Male	21	Mum	EOD	3	Youngest of 5 children. 2 siblings are his mother’s from a previous marriage; 2 from his fathers.	Lives away.	Postgraduate education.
Jade	Female	16	Dad	FTD	1	Jade and Amy are sisters, although Jade is adopted. Amy has a young daughter.	Home.	Education
Amy	Female	24						Employed
Austin	Male	8	Dad	FTD	4	Austin has two siblings from his mother’s previous marriage so do not share the same father	Home	Education
Ava	Female	22	Mum	FTD	2	Oldest of two siblings	Home	Postgraduate education
Holly	Female	21	Dad	FTD	4 Deceased less than six months.	Oldest of two siblings	Away	Employed
Rhian	Female	24	Mum	Early onset dementia	5	Youngest of two siblings	Lives at home with parents	Employed
Harriet	Female	17	Dad	Vascular dementia	1	Only child; divorced parents	Lives with her boyfriend, near her father.	Education
Nathan	Male	22	Dad	FTD	1	Youngest of two siblings	Lives away at university	Education

## Findings

Inevitably perhaps, given the invitation to tell the story of their parent’s dementia and also in light of the sorts of plots, scripts and timeframes people living in western society tend to use to recount and explain their lives and the things which happen to them (Booker, 2006; Connelly & Clandinin, 1990; Freeman, 2014; Goodson, 2013), people usually began by invoking hindsight (Freeman, 2010). Their narratives then linked events, perceptions, and experiences in an attempt to make, and bring, some personal sense to their parent’s dementia journey. Here, our focus is on young people’s stories around: realizing something was “wrong” with their parent; navigating an illness that characteristically entails an unpredictable trajectory and messy timescale; and, the consequences for their own lives. Unfortunately, we only have space to share a fraction of the stories dealing with these themes.

### Something Is Amiss: “You Don’t Normally Put Three and a Half Spoons of Sugar in Your Tea”

When the young people began their stories, they almost all recalled some behavior or incident—which by the time of the narrating had come to be imbued with epiphanic status—that suggested that “something” was not right. This “something” was rarely associated with the possibility of dementia: it was, however, unusual and represented a change:

Dad started taking days off work and I knew something was going on and he basically just started to get really miserable, he got upset and I’d never seen him crying or anything before so I thought something’s going on. He got upset over losing car keys. (Evie)

She was just very, very different. Like she became very social withdrawn, whereas before, she worked for the BBC … she wrote the news … She’d been very competent and intelligent and quite well respected in her job like, I’ve always been incredibly close to my mum, she’s always been my best friend, especially as my parents were divorced … and then we started arguing and we’d never really argued before … she’d be interested in what I had to say but she’d give me a lot less back or then she’d forget words, jumble her words and I’d get really frustrated. (Madison)

He always had 1 and a half teaspoons of sugar in his tea and I remember watching him put three and a half in his tea and thinking you don’t normally put three and a half spoons of sugar in your tea … then he forgot my birthday. (Gabrielle)

In hindsight that we look back and we can sort of pick up a few things that we’ve noticed about Dad and at the time it didn’t seem much at all, it just seemed maybe a bit odd or almost normal in some respects, he was going around bookshops and charity shops and things and looking for cheap books, sounds really simple but … we went on holiday in Dorset and every time we went into Lyme Regis, he’d always want to go into the same shops every time, even if it was the day after, we’d go in the same shop to see if they had something in there. (Nathan)

Changes—some extreme—in parenting behaviors were also experienced:

He’d started to do really odd things where I’d go to Tesco with him … and I’d look at something and go “oh look there’s that new Kindle tablet out” and he’d go “Do you like it?” and I’d go “yeah” and he’d go “we’ll have this please” at first I thought this is GREAT. (Evie)

At the start it was strange things like, it sounds silly but when I was about 16, she would buy me cigarettes and alcohol and that wasn’t normal, that was strange. (Colin)

Interests changed too:

She’s always loved Coronation Street and she got less and less interested … until eventually she was just sort of a good few years ago “oh there’s too many new people in it” or “the story lines move too fast” and the same … she’d always just absolutely loved reading and then she sort of found books more and more complicated and I like go back home now and I find books that she started, I don’t know, maybe seven or eight years ago, and she’s underlined characters in the first chapter so she can try and look back on them. (Elizabeth)

He bought, it’s like a sailing canoe. It was a really rash purchase, it was really expensive. It was like seven and a half grand. And he’s never spent that much money on anything brand new … But then he just set his heart on this boat and he wanted it and it’s really expensive, we’re not gonna use it that much.but he just went ahead and bought it. We got it delivered, it was like “great, we’ve got a boat” … it was just abnormal. (Alex)

As we have noted, such incidents and behaviors only acquired meaning as the condition progressed and with the benefit of hindsight and a diagnosis. Initially, it was not clear what was amiss and dementia was not in the frame. This was clearly a confusing time with various possibilities obscuring the picture. For instance, the young people questioned whether behaviors were indeed new, wondering if their parent had always been like this, and they were only just getting old enough to notice:

Looking back, it was quite difficult to tell at first. You put things down to sort of little eccentricities. (Beth)

The problem that I’d found looking back now is … when she was developing it I was getting older and so I thought “well maybe this is actually her personality, so she’s maybe always been like that and I haven’t noticed. Maybe she’s always been a bit flappy and I haven’t noticed,” and actually when I was at University I thought “oh, maybe she’s not as clever as I once thought she was and because I’m now maybe at a different intelligence level.” (Lily)

Lily went on how to describe how she learned her Mum’s behavior was different to other parents’ when she compared her with her boyfriend’s mother:

[We] went travelling to America from July to October … Mum was okay then, but like I remember James’ Mum was checking up on us all the time, Mum was very blasé about it, I think I rang her once in the whole thing and she’s like, I was like “I’m really sorry I haven’t called you” and was like [upbeat] “oh it doesn’t matter, if anything happened I’m sure someone would tell me,” didn’t really seem worried about my safety. (Lily)

Additionally, a host of other factors meant that there were other plausible explanations for changes in behavior. Social changes such as redundancy, divorce, and the children having left home were each taken into account at the outset:

She really filled the role of parent and vicar’s wife. She would always do all the flower arranging, all that sort of thing. And then my Dad actually retired, five years ago, and that’s when we noticed, that’s when things started out of control, where she was a little bit ditzy, she was becoming really unaware of what was happening around her … I came to the conclusion it all happened around the same time. I was the final child to leave home which meant she was no longer the mother, and then Dad stopped working which meant she was no longer the vicar’s wife so she was kind of left without a purpose. (Colin)

Similarly, medical causes, such as stress, depression, and the menopause were also considered potential explanations. Parents also went to particular lengths to conceal their symptoms from their children and deny the extent of problems:

I’d noticed she’d been forgetting things for quite a while, like she’d been depressed when I was a bit younger so I thought maybe it was just that or like menopause and that’s what I was kind of praying for … Before she was diagnosed she kept refusing to accept there was anything wrong so I got very, very frustrated and was like “why won’t you just go?” and then eventually she was like “I’m scared” and I was like, the sooner you find out what it is, the sooner you can get help. (Madison)

Concealment was further enabled when children were not living with their parents. Sheena, who had been 21 and in her final year of University in another town described how she was left in the dark by her mother during her father’s diagnosis:

I was quite sheltered from it … my mum’s quite closed about things, she sees it as her job to look after my Dad and you know she doesn’t feel like I need to know things like when he goes into hospital or things like that whereas I kind of do, so she’s trying to protect me so I see why she does that. So initially when he was diagnosed, I knew there was something wrong because my parents were arguing quite a lot, my Dad’s behaviour was really bizarre, really unusual … I was going back to University for my last year so I knew something was wrong but I wasn’t 100% sure what was going on … I’d probably say potentially a month or two after she really knew because my Dad was so erratic that it couldn’t really be hidden. (Sheena)

Alex described how he and his sister had difficulties identifying actually which parent was experiencing problems as they were each living away:

We’re all scratching our heads like, “is Mum alright?” and maybe there is something wrong with Mum as well … I’d be like, “I don’t know, there’s something wrong but I don’t really know what it is” … Like Mum was part of it as well because they’re always together, then and the way that they interact, like the way that they don’t help each other when they interact. (Alex)

Nevertheless, over a period of time, the catalogue of “odd” behaviors grew. Symptoms became harder to ignore, and medical attention was subsequently sought. In some cases, the young people played a part in precipitating consultation and diagnosis. For example:

When I’m 19, I go in for a blood pressure check because I was on the pill, 18 maybe. And I just said “oh my Dad keeps putting the kettle on without any water in.” And that started an assessment that came out as anxiety or depression because he’d lost his job in between that time. (Rachael)

For Austin, the fact that, then aged 4, he was able to build Lego when his father could not prompted a visit to the doctor:

I think it was when he couldn’t do the Lego because I would sit with him and he would pick up the wrong pieces and didn’t know what to do and he just kept on sighing, saying “I just can’t do it.” (Austin)

Family biographies which included previous experience with dementia were also important:

My Granny had dementia, though she’s not related … my Mum started forgetting things … I was like “you’re just like Granny.” (Madison)

I was just about to take my GCSEs at that point and before that I think she’s got an older brother John, who remembered their Dad going through the same thing and he’d flagged stuff up a few years before even … I was about 13 because he’d noticed the really early signs, where I just thought “oh she’s just a bit eccentric.” (Beth)

## Navigating the Pathway

YOD diagnosis can be a lengthy process involving much uncertainty as other possibilities are ruled out:

It took ages. I think the first things was we had an appointment with the GP to talk about it and I think we all went in like as a family and so me, Mum, my Dad, my Uncle even came up … there was that appointment … it felt weird because we were all sat talking about my mum as if she wasn’t there … a few months after that they referred her for psychiatric … I think I drove her to a psychiatrist appointment and I sat in with her on the appointment and again the psychiatrist was just asking loads of really quite kind of complicated questions that would need a complicated answer and my mum at that stage was just giving sort of, she couldn’t really take anything seriously, or I think she was kind of avoiding the questions … then a few months later I think then she must have had an MRI scan and then a few months later I think then we got a diagnosis. (Beth)

I think we all just thought it was depression for a while based on him losing his job … He got banned from driving and started the diagnosis process but it took seven, eight months to get a full diagnosis. Really long. We were like JUST TELL US! It came out Alzheimer’s Disease, not related to Vascular Dementia because we all thought “oh it’s the aneurysm” (Rachael).

A year into investigations, Alex described the working diagnosis as “mushy middle ground.” He recalled a hospital appointment where he had expected some explanation for his father’s condition. In fact, it would be another year before a formal diagnosis was reached:

We were expecting something to happen, like them to say you’ve got this, this and this, this is this, this is gonna happen, this is how long it takes, this is what we know, and this is what we can do about it, or well, we can’t do anything about it, but this what’s gonna happen.

Over the diagnostic period, life goes on involving changes, such as going to university, which while “normal” can also be unsettling. A parental dementia diagnosis can exacerbate stress and turn worlds upside down:

The first 18 years of my life, I’d lived in Sunderland, gone to the same school, lived in the same house and then when I was 18, just everything just turned upside down, obviously my Mum had this diagnosis, we moved everything down South and then a week later, I was off to Leeds to Uni. (Beth)

Nathan, found his father’s diagnosis, combined with making university arrangements overwhelming:

Things were deteriorating. It was a pretty huge shock to me. Things started to go pretty downhill for myself. During the summer, the same time we got the diagnosis, that was the same time I sent off my application for my degree. Great timing right? Not … I’d got confirmation that I was on my course but accommodation hadn’t quite gone through … I thought I’d missed the boat with accommodation and everything with my Dad … I tried to end things for myself if you know what I mean by that.

Jutel (2009) has described how diagnosis, the process of naming and categorizing, has conferred enormous power and authority on the medical profession. This is not least because: “diagnosis, as both category and process (Blaxter, 1978), is infused with all manner of things, social, historical, cultural … Diagnoses are embedded in social relations and serve as the basis of identity formation and management” (McGann & Hutson, 2011, pp. xxix–xxxi).

Diagnosis usually leads to changes for the patient and their families. It may provide an explanation for symptoms, lead to decisions regarding treatment and offer a prognosis and implicate social expectations: being ill, for example, exempts one from certain obligations (Parsons, 1951). However, initial uncertainty was hard to bear and entering into the diagnostic process does not immediately clarify the situation, especially with YOD where other explanations are usually offered at the outset.

Having received a diagnosis, reactions were mixed. Although a dementia diagnosis was expected, some were shocked when it became definitive. Additionally, as dementia is a symptom of a number of diseases, a more precise conclusion would be reached further down the line which would predict or offer an explanation for particular behaviors associated with particular variants.

Strange because I’d kind of already come to the conclusion that it was dementia but when I heard it was horrific. When I heard the news, it was the only time when I actually externally sort of had a reaction, where I got off the phone and immediately I started crying and I think that’s going to happen to anyone. (Colin)

Of course, obtaining a diagnosis did not change the gravity of the situation, as Beth stated: “I mean it didn’t actually move forward really, you’d think it would have but it kind of just went back to normal.” It did, however, provide some answers, and there were positives. There could be a sense of relief in having an explanation, and a diagnosis was helpful in securing support and offering exemption from social roles and expectations.

I was kind of relieved … the initial diagnosis was more important than the final one, we’d been waiting for it for a while really, at least a year, to start the process so the initial one was a big relief and it meant that my Mum [the carer] could claim loads of support and we could start being active whereas before we were all being passive and not doing anything, sort of like the moment when people started doing things. (Rachael)

She had to stop work … They gave her six months sick leave while the diagnosis was being had and then they put her on her pension. (Ava)

The diagnosis could be beneficial to parent–child relationships. Dementia discourses, especially “personhood” (Kitwood, 1997) demand that we separate the illness from the person. However, until a diagnosis is confirmed, the children of persons with dementia are somewhat left at sea, as was the case for Grace, who was 11 when her Mum received her diagnosis:

I thought she should be my carer, looking after me but I didn’t know she had dementia then and I just thought she was being silly. (Grace)

Actually when she had a diagnosis for it I was like “okay, well it’s not actually her fault” and so because of that it was then a lot easier to deal with not just for me … You’d just want to grab her and be like “why are you doing this, why are you acting like this?” but then what would be even more annoying … she would just giggle and just find it hilarious … you were like “please just take me seriously” but you don’t realise that she couldn’t take anything seriously. (Beth)

The involvement of the medical profession also absolved the young people of particular responsibilities. Driving, for instance, was often a serious worry:

Driving, that’s a tough one because he really loves it, he’s like, I don’t wanna stop driving. I wouldn’t wanna discuss it with him, I know that that’s a subject that would touch a nerve so I just have to wait for some medical professional to tell him that he can’t drive and then that saves us from stopping him driving. (Alex)

Having a label also made young people more inclined to disclose the situation to others because they had something concrete to tell them, and this had the potential to generate greater support, as Rachael continued.

It’s also the moment we told my Dad’s sister and it’s hard for her because she lives in Brighton and at that point, she was only coming up once a year, she now comes up every other week … because we have my Nan to look after as well. (Rachel)

I didn’t know what was wrong, I couldn’t exactly even if I had had the guts to tell my friends, I wouldn’t have been able to know what was wrong to tell them. (Madison)

However, it is the case that dementia continues to be a socially stigmatizing (Goffman, 1963) and identity spoiling condition (Sabat, Johnson, Swarbrick, & Keady, 2011; Werner, Goldsteing, & Buchbinder, 2010), and it is possible to be stigmatized by association with implications for personal and familial identities, sense of self and how others view and relate to the young people who have a parent with dementia. This theme is explored elsewhere (Hall and Sikes, 2018).

## The Unpredictability of Dementia

The narratives shared here tell of confusion. Whereas, traditional illness stories are frequently characterized by a catastrophic event or diagnosis (Frank, 2013), dementia narratives, like those concerning other degenerative conditions with uncertain trajectories such as Parkinson’s disease (Barken, 2014), see an initial incident followed by periods of stability, if not normality. The young people began by sharing anecdotes that became more meaningful as the story progressed. This is not a straightforward linear process. Young people are witnessing their parents’ health decline, in an unpredictable fashion. There may be “settled” phases, but these are followed by decline.

You think you’ve got it nailed, you think, “yeah great, I’ve really got this Alzheimer’s thing, great” and then yeah … when I saw you last, Mum had taken a massive turn for the worse and I think since then, it’s been a wobbly plateau so the next step down hasn’t happened yet which I think means you get time to recover in your head, you go through the grief cycle everytime. (Elizabeth)

At the moment, I’m in more of like a settled phase of it but I feel I always settled, I get used to it and then she gets that bit worse and then obviously I get really upset again. When she gets worse, it’s more at the forefront whereas now I’m sad but it’s not dominating my life whereas when she gets a bit worse, it’s more noticeable then I get sad about it again … you get used to a new normal every few weeks it seems, or even a shorter time, there’s a new normal and it’s so unpredictable. (Madison)

One of the most notable misconceptions about dementia is a lack of understanding that it is terminal. Young people are forced to confront the realization that it would lead to their parent’s death with implications for their own biographies:

I think the thing that stood out for me was that they said she had about 7 years left and at the time I think that was just like I couldn’t believe I was having to prepare myself for my Mum … as soon as I heard the 7 years I remember being in tears looking back, actually 7 years would have been fantastic because it was only 3 in the end. (Bethany)

It just doesn’t sit with me at all. I don’t want to accept this is happening or the consequences of it … we were told less than 3 years, I think that would have been 3 years from the signs, knowing that we are effectively a year on, you do wonder what is left, what time do we have left. I can’t accept it, I don’t understand it … why has this happened to me and my family, what’s my Dad done to deserve this? (Nathan).

They revealed that how they spent time with their parents in the present while they could and talked of grieving for lost futures:

I go back every holiday just to make sure she remembers my face really … I’ve got one brother who lives all the way down South … he’s the one who Mum struggles the most to remember … I keep going back to make sure that doesn’t happen. (Colin)

The worst part is, that cuts me up, he never saw me graduate, he’s never gonna see me get married, never gonna see me have children. That’s what gets me (Holly).

The unpredictability of dementia renders planning difficult. And indeed, there is much to plan: young people’s own lives, care arrangements for parents, and financial considerations:

You just don’t know what the next step is going to be or whether she’ll start or how quickly it will come on and things … I think Mum in a home, she wouldn’t really notice but it’s just whether it’s right for her, but no I’ve no idea really how to plan. (Lily)

If I knew from the beginning, if they’d said it was going to be a year, I would have gone home … with dementia, you don’t know when the end is going to be the end … this could be the last year, the last month … there could be another three years. (Sheena)

This temporal uncertainty had consequences for the young people’s future biographies.

## Discussion

Our study has shown that children and young people who have a parent with dementia experience the lengthy diagnostic period in similar ways to adult carer (Perry-Young et al., 2016; van Vliet et al., 2011). However, in line with the limited evidence suggesting that parental dementia in families with children and young people causes greater social and psychological upset, damage, tension, hardship, and family break up than it does in families where children are grown up, our findings also suggest that for young people starting out on their lives, the process of coming to diagnosis and then negotiating life with a parent with dementia can be particularly difficult, influencing life choices, occasioning guilt, and bringing considerable stress and unhappiness. It impacts their biographies in significant ways requiring them to make sense of what has happened to them as best they may.

Of course, serious and terminal illness is always going to be distressing, painful, and upsetting and, perhaps, especially so when it comes prematurely. YOD is clearly premature both for the person with the condition and for their relatives and friends. Bury’s (1982) notion of “biographical disruption,” originating in a study of rheumatoid arthritis in people who were relatively young to develop that condition, is useful for considering the biographical and interpretational work the young people taking part in our study engaged in. Bury’s study demonstrated that events occurring out of time in terms of age and stage of life course are likely to result in significant disruption, affecting relationships, and day-to-day life (Lawton, 2003; Pound et al. 1998; Sanders et al., 2002). This is significant for young people who have a parent with YOD, as this is decidedly not a biographically anticipated event (Williams, 2000), and there are, as yet, no or few, social scripts or templates (Goodson, 2013) or culturally sanctioned plotlines (Freeman, 2010) to help them make sense of what is going on (Sikes & Hall, 2016).

Notions of time and of what constitutes normal temporal progression are profoundly important here. As we have seen from the stories we were told, making sense of and adapting to parental dementia draws heavily on giving temporal meaning to events and situations. Thus, and for example, hindsight (Freeman, 2010) helps to give meaning to events which were initially nonsensical, while a diagnosis provides an explanation for what has happened to date which also has present and future social currency in that expectations concerning roles, relationships, behaviors, attach to particular diagnoses. Having said this, and as we have noted, there can be misconceptions, particularly around the idea that dementia is a disease of older people.

Hindsight and diagnosis play significant roles in the process of narrative reconstruction (Williams, 1984) from being a “regular” young person to becoming a young person with a parent with dementia. Williams suggests that in the face of illness and following a medical diagnosis, the reconstruction of one’s personal narrative can help to “reconstitute and repair ruptures between body, self, and world by linking and interpreting different aspects of biography in order to realign present and past and self and society” (1984, p. 197).

This reconstruction is not, however, a straightforward process and contemplating how it might work for these young people leads us to a fundamental problem in making sense of the unpredictable illness trajectory that dementia involves. Mead (1932, 1934, 1938) asserts that time is not experienced in a linear fashion, that the past, present, and future are not understood in isolation. Freeman similarly talks of the way in which “clock time is but one concept of time … which does not do justice to those non-linear aspects of human time (in this case, memory and sense making) that go back and forth, forth and back” (2010, p. 178). Research by Barken (2014) focusing on Parkinson’s Disease, which is also to some extent unpredictable, has indicated that biographical and time work is undertaken with the past, present, and future being reinterpreted to achieve biographical continuity and a sense of self. This was the case for the dementia narratives, with young people keeping the future in mind in the present and using the past to make sense of the present. In other words, people have to use their imaginations when constructing and reconstructing personal narratives and, as Andrews puts it, coupling narrative and imagination “brings into focus: (1) the salience of the dynamic nature of the temporal; (2) a mediation between the real and the not real; and (3) the complexity of the construction of ‘the other’” (2014, p. 2).

The complexity of “the other”—the parent with dementia, plus the multidimensional nature of dementia, complicates the biographical work further. The excerpts reveal the relentless nature of the disruption. Lives are disrupted when a parent displays odd behavior. They are disrupted when the diagnosis is reached. They are disrupted when a parent loses the ability to recognize their child or speak with them. They are disrupted when their parent dies. Indeed, where dementia is concerned, families can be understood to experience what has been described as a social and biological death (Sweeting & Gilhooley, 1997).

Additionally, dementia in a young person’s biography does not necessarily end with the parents’ death. Williams (2000) suggests that illness is not always a biographical disruption, suggesting that some people do anticipate illness in their own lives (Hubbard, Kidd, & Kearney, 2010). Indeed, many participants indicated that they had a fear of developing dementia themselves even without evidence their parent had a genetic form.

Baldwin (2006, 2016) has written about the tyranny of narrative and how people with dementia (and mental illnesses) can experience a strong sense of social exclusion which affects their identity, their sense of self because their stories frequently lack the sort of narrative coherence expected in our society. This is the case for these young people too.

Children and young people who have a parent with dementia usually have to cope with a chaotic and unpredictable situation where, even prior to diagnosis their mum or dad begins to change and becomes more and more “other.” Diagnosis can be seen to formalize this otherness which, as the disease progresses, becomes ever more marked and extreme leaving, in most respects, little vestige of the parent who was. Death is the inevitable conclusion and for many of our respondents, both those who were anticipating and those who had experienced it, it was seen as a “blessing,” and a release for both parent and child.

In undertaking this project, a primary aim was collect and share narratives which could provide young people facing parental dementia with potential scripts, templates, storylines that they could (or not) identify with and which could offer support—sometimes through providing permission to feel certain emotions—as they navigated their parent’s illness. Theirs is usually a difficult, painful, and disruptive journey with potentially serious implications for life chances and for emotional and mental wellbeing. The data revealed biographical work in the form of narrative review and reconstruction seems to offer an important and helpful personal resource. Indeed, some of our participants said that they found taking part in the study itself helpful in this respect. We suggested elsewhere that counseling should be built in to support systems provided for families living with young onset dementia. Here, we want to suggest that narrative therapy (White, 2007; White & Epston, 1990) could be particularly useful in circumstances where, as Alex describes, “every time I see him he’s the worst he’s ever been and the best he’ll ever be.”
